# Fine Mapping of QTLs for Alkaline Tolerance in Crucian Carp (*Carassius auratus*) Using Genome-Wide SNP Markers

**DOI:** 10.3390/genes15060751

**Published:** 2024-06-07

**Authors:** Liang Zhang, Baofeng Su, Jing Huang, Limin Zhang, Yumei Chang, Guo Hu

**Affiliations:** 1 Key Laboratory of Freshwater Aquatic Biotechnology and Breeding, Ministry of Agriculture and Rural Affairs, Heilongjiang River Fisheries Research Institute of Chinese Academy of Fishery Sciences, Harbin 150070, China; zhangliang@hrfri.ac.cn; 2 Key Laboratory of Fish Stress Resistance Breeding and Germplasm Characteristics on Special Habitats Heilongjiang Province, Heilongjiang River Fisheries Research Institute of Chinese Academy of Fishery Sciences, Harbin 150070, China; subfeng@auburn.edu (B.S.); huangjing@hrfri.ac.cn (J.H.); zhanglimin@hrfri.ac.cn (L.Z.); 3 School of Fisheries, Aquaculture and Aquatic Sciences, Auburn University, Auburn, AL 36849, USA

**Keywords:** crucian carp, genetic linkage map, QTL mapping, alkali-tolerant fish

## Abstract

Crucian carp (*Carassius auratus)* is widely distributed in the world and has become an economically freshwater fish. The population in Lake Dali Nur can tolerate the extreme alkaline environment with alkalinity over 50 mmol/L (pH 9.6), thus providing a special model for exploring alkali-tolerant molecular markers in an extremely alkaline environment. In this study, we constructed a high-density and high-resolution linkage map with 16,224 SNP markers based on genotyping-by-sequencing (GBS) consisting of 152 progenies and conducted QTL studies for alkali-tolerant traits. The total length of the linkage map was 3918.893 cM, with an average distance of 0.241 cM. Two QTLs for the ammonia-N-tolerant trait were detected on LG27 and LG45. A QTL for the urea-N-tolerant trait was detected on LG27. Interestingly, mapping the two QTLs on LG27 revealed that the mapped genes were both located in the intron of CDC42. GO functional annotation and KEGG enrichment analysis results indicated that the biological functions might be involved in the cell cycle, cellular senescence, MAPK, and Ras signaling pathways. These findings suggest that CDC42 may play an important role in the process of dealing with extremely alkaline environments.

## 1. Introduction

Alkalization and salinization, which are serious threat to the development of inland lakes and freshwater fishery resources, are occurring under both global warming and human activities. Usually, the pH of these alkaline and saline lakes is higher than 9.0, but some fish can survive naturally in alkaline and saline lakes. Crucian carp (*Carassius auratus*) (2*n* = 100) is widely distributed in major watersheds and lakes around the world and has become an important economic freshwater fish species for aquaculture in more than 100 countries [1]. Crucian carp products have become an important source of daily protein for many people, especially in some food-poor areas [2]. Lake Dali Nur (116°25′–116°45′ E, 43°13′–43°23′ N), located in the eastern Inner Mongolia Plateau in North China, is a barrier lake formed by volcanic eruptions. Because the evaporation is less than the precipitation and inflow, Lake Dali Nur has become a typical saltwater lake with a high level of alkali carbonate. As one of the most extreme aquatic environments on Earth, the alkalinity of this lake can reach up to 50 mmol/L (pH 9.6) [3]. One example of the organisms in this extreme environment is the Lake Dali Nur crucian carp, which can not only survive in the water with high levels of alkaline carbonate but also reproduce and grow to the point of becoming one of the few remaining fish species [4]. Alkalinized and saline-alkalized waters can disrupt the acid-base balance and inhibit the excretion of nitrogen-containing wastes, which are fatal to the growth and reproduction of freshwater fish in these extreme environments [5]. However, Lake Dali Nur crucian carp can naturally survive in such extreme environments and have become an important source of income for local natives living around Lake Dali Nur. At the same time, during the annual migration and spawning season, the Lake Dali Nur crucian carp is also an important food source for migratory birds [6]. Although they play an important role in local fishery development and ecological stability, its mechanisms of adaptation to alkaline stress environments and high tolerant for alkalinity are not well understood.

Ammonia is the main metabolic end product of proteins or amino acids that cannot be digested and absorbed by the body [7,8]. It is toxic to all fish and must be detoxed or excreted. Therefore, timely and effective ammonia excretion is an important mechanism for maintaining physiological homeostasis in animals [9]. Ammoniacal metabolism is the main strategy of most freshwater fish, in which ammonia is excreted as nitrogen waste through the gills, and excess nitrogen is metabolized and eliminated as urea [10]. However, under high-alkaline conditions, H+ capture short-circuits, reducing or reversing the NH_3_ diffusion gradient in the normal “blood-water” circulation of the fish body, leading to a large accumulation of ammonia in plasma and tissues, causing ammonia poisoning and even death [11]. Ammonia poisoning has been shown to be the main reason why some fish cannot adapt to high-alkaline environments [12,13]. Therefore, efficient ammonia excretion and ammonia tolerance mechanisms may be important strategies that allow Lake Dali Nur crucian carp to adapt to high alkaline environments.

Genetic linkage map is an important tool for the study of genetics and genomics in the quantitative trait locus (QTL) mapping [14,15]. In the past few years, researchers have constructed a large number of fish genetic linkage maps and used SNP markers to explore QTL maps for important economic traits, such as growth-related and disease traits, which provide important strategies for molecular breeding [16,17,18,19]. Through the study of alkalinity gradients, researchers have revealed the toxic effects of different alkali water quality conditions on Lake Dali Nur crucian carp [20,21]. These findings indicated that the Lake Dali Nur Crucian carp has unique genomic adaptability to cope with extreme alkaline environments. In addition, previous studies have shown that the expression of alkali-tolerant genes Rhag, Rhcg1, Rhcg2, and Rhbg is significantly upregulated in Lake Dali Nur Amur ide (*Leuciscus waleckii*). However, the alkali-tolerant genes of Lake Dali Nur crucian carp have not been well studied [22]. Therefore, studying the genetic linkage map and QTL mapping of Lake Dali Nur crucian carp genome would be helpful for comprehensively exploring and understanding the potential molecular mechanisms underlying its adaptation to high alkalinity.

In this study, a high-density linkage map with SNP markers was constructed. Subsequently, accurately mapped QTLs for alkali-tolerant traits in the crucian carp and efficient ammonia excretion and nitrogen tolerant genes were also identified. In addition, the possible biological functions of the candidate genes were analyzed by GO functional annotation and KEGG enrichment analysis. The findings of this study should be valuable for a comprehensive exploration and understanding of the genetic architecture for extreme alkalinity adaptation, as well as potential breeding applications for crucian carp in Lake Dali Nur.

## 2. Materials and Methods

### 2.1. Mapping Family and Phenotypic Measurement

To enhance the likelihood of detecting QTLs associated with alkaline tolerance, the hybrid population were used to combine the diverse genetic backgrounds of wild-type crucian carp in Lake Dali Nur and domesticated bred red crucian carp. Thereby, the increased genetic variability present in hybrids provided a broader genetic base for QTL detection. In this study, the maternal parent fish used in the experiment was wild Lake Dali Nur crucian carp, and the paternal parent fish was artificially bred with freshwater red crucian carp. Three thousand (3000) F1 hybrids were obtained, which were raised in the Hulan Aquaculture Experimental Station of the Heilongjiang River Fisheries Research Institute, Harbin, China. A total of 180 F1 hybrids were randomly assigned to 6 identical aquariums and raised under the same feeding conditions with standard feeding procedures.

NaHCO_3_ was added to each aquarium in equal amounts at a gradient of 20 mM/L per day. At the seventh day, hybrid F1 began to die. Then, the alkalinity was maintained, the cumulative death rate of 96 h was more than 50% as the benchmark, and the subsequent surviving individuals were regarded as alkali-tolerant individuals. During the alkalinity tolerance test, a small amount of blood of the deceased and surviving individuals was collected for the determination of ammonia-N and urea-N contents, which were used as physiological indexes to measure the alkali tolerance traits according to the kit instructions provided by Nanjing Jiancheng Technology (C013-2-1, Nanjing Jiancheng, Nanjing, China; A086-1-1, Nanjing Jiancheng, Nanjing, China).

### 2.2. DNA Extraction

A small number of fin strips were cut and placed in a centrifuge tube. The genomic DNA was extracted from preserved fins using an Invitrogen DNA Kit according to the manufacturer’s experimental protocol. The DNA concentration was determined using a NanoDrop 8000 spectrophotometer (Thermo Fisher Scientific, MA, USA), and the RNA integrity was checked by 1% agarose gel electrophoresis. The qualified DNA was stored in a −20 °C freezer.

### 2.3. Library Construction and Sequencing

Genomic DNA (0.1–1 μg) and restriction endonuclease MseI (#10149200, New England Biolabs, Ipswich, MA, USA) were added to the centrifuge tube for digestion at 37 °C. T4 DNA ligase (#M0202, NEB, USA), ATP, and Y-adapter N were added; the ligation system was allowed to react overnight; and then the enzyme was inactivated at 65 °C. The restriction endonuclease NlaIII (#R0125V, NEB, USA) was added to the ligation reaction, which was subsequently digested at 37 °C for 1 h. After the digestion reaction, Agencourt AMPure XP (#A63882, Beckman, Bremen, Germany) was used for purification. PCR amplification was performed on each purified sample with Phusion Master Mix (#M0532S, NEB, USA) using common primers and index primers. All DNA samples were mixed, and 2% agarose gel electrophoresis was performed. A gel extraction kit (#28704, Qiagen, Valencia, CA, USA) was used to extract 425 bp to 450 bp fragments. After purification by Agencourt AMPure XP (#A63882, Beckman, GER), double-terminal sequencing was performed using the Illumina HiSeq sequencing platform.

### 2.4. SNP Extraction

All individual sequences were compared with the *Crucian carp* chromosomal level reference genome (NCBI RefSeq assembly: GCF_003368295-1) using BWA software (version 0.7.17) [23]. The sequence index was first established by the BWA index, and then the double-ended sequence comparison was performed by BWA-MEM. The SAM files were converted to BAM files using SAMtools (version 1.9.0) [22], and reads that were not aligned to the reference sequence were removed. The mpileup command of BCFtools (version 1.17) [24] was used to sort the BAM files and generate the BCF files. The call command of BCFtools was used to detect the mutation site of the BCF file and generate the VCF file. Using the setting QUAL > 20 and DP > 5 and the filter command of BCFtools, the mutation sites were filtered. The genotyping data of 100,895 SNPs in 154 fish samples were obtained, which were used to study the subsequent relationship between QTL peak and genotype.

### 2.5. Linkage Map Construction

Lep-MAP3 software (version 0.7.17) [25] was used to construct the genetic linkage map group. The SNP data were entered into the core module, ParentCall2, to call the missing or incorrect parental genotypes. Next, the Filtering2 module was used for data filtering. Generally, the bias separation (datatolerance parameter) was set to 0.01, the integrity (missingLimit parameter) was set to 0.1, and the minimum allele frequency (MAFLimit) was set to 0.05. Then, the SeparateChromosomes2 module was used to debug the chain group, the LodLimit was adjusted from 5 to 20, the threshold size of the number of markers for each chain group was set to 20, and an LOD value of 10 and a size of 50 were selected as the parameters for separating the chain group. The JoinSingles2All module was used to encrypt the linkage groups, reduce the LOD threshold for partitioning the linkage groups, and select an LOD value of 9 to recalculate the LOD values of the tags. Then, these undivided tags were assigned to the appropriate linkage groups again. After merging the individual tags, the OrderMarkers2 module was used to sort the tags in each linkage group. We ran the OrderMarkers2 module 50 times for each linkage population and selected the order with the highest likelihood value as the final order. The Kosambi function was used to calculate the genetic map distance, and LinkageMapView was used to construct the genetic map.

### 2.6. QTL Mapping

The Rqtl software package (version 1.66) [26] was used to analyze the alkali-tolerant traits. The same SNP molecular markers were removed from the genetic map, and the trait was scanned using multiple imputation. QTL scanning was performed using the default model, and the significance of the genome-wide LOD threshold was detected by a permutation test (1000 times). The obtained QTL was modeled by makeqtl; the location, LOD, additive effect value, and dominant effect value of each QTL were obtained by fitqtl, and the 90–95% Bayesian location interval of each QTL was obtained by lodint.

### 2.7. Candidate Genes Analysis

The Rclusterprofiller software package (version 4.3.3) [27] was used to analyze the GO functional annotation and KEGG pathway enrichment. The Cytoscape software (version 3.9.0) [28] was used to analyze the Interaction network of potential alkali tolerance proteins.

### 2.8. Statistical Analysis

Statistical significance was tested by the *t* test, with mean ± SD *p*-value was used to represent the significance of statistical results. Statistical analysis was produced by R software (version 4.3.3).

## 3. Results

### 3.1. Summary of Sequencing Data

We sampled and measured 180 offsprings from the full-sib family, 28 of which were removed due to DNA degradation or low sequencing depth. Finally, 152 progeny and the 2 parents were used to construct linkage map, and QTL analysis was performed. Overall, we sequenced the original reads of 57.540 gigabits. The sequencing quality Q20 ≥ 90% and Q30% ≥ 85%, GC distribution of samples was normal. The quantity and quality of the database met the requirements of genetic linkage map construction.

### 3.2. Phenotypic Data

Alkali-tolerant traits are summarized in Table 1. The ammonia-N and urea-N ranged from 0.827 to 14.903 mmol/L and 0.143 to 1.308 mmol/L, with average values of 6.856 ± 0.093 mmol/L and 0.681 ± 0.166 mmol/L, respectively. The Phenotypic Pearson correlation coefficient was r = 0.752. The coefficients of variation (CVs) of ammonia-N and urea-N were 0.418 and 0.547, respectively.

### 3.3. Linkage Map Construction

**Figure 1:**
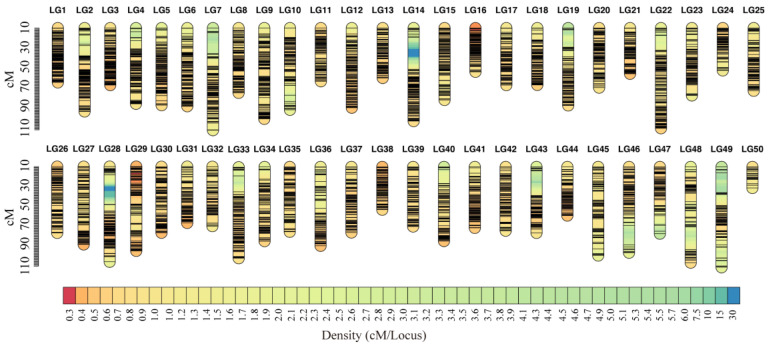
The high-density linkage genetic map for alkaline tolerance traits mapping.

We analyzed the sequencing SNPs with a LOD threshold of 20 and found that 16,224 SNPs were screened to 50 linkage groups. The linked groups were sorted using the OrderMarker2 module labeled with different information types, and then the order with the greatest likelihood value was selected as the final order. Finally, 50 linkage groups containing 16,224 SNPs were constructed (Figure 1). The total distance was 3918.893 cM, and the average distance was 38.696 cM. The spacing distance of each linkage group was 0.085 cM (LG1)~0.729 cM (LG49), and the average spacing distance was 0.241 cM. The largest linked group was LG7, containing 462 unique SNPs with a length of 117.347 cM, and the smallest linked group was LG50, containing 36 unique SNPs with a length of 23.209 cM (Table S1).

### 3.4. QTL Mapping

**Figure 2:**
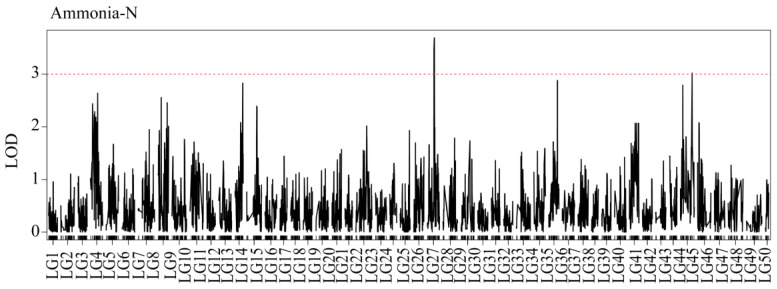
QTL mapping and significant regions were identified for the concentration of ammonia-N. The red dashed line represents the delimited LOD threshold equal to 3.

**Figure 3:**
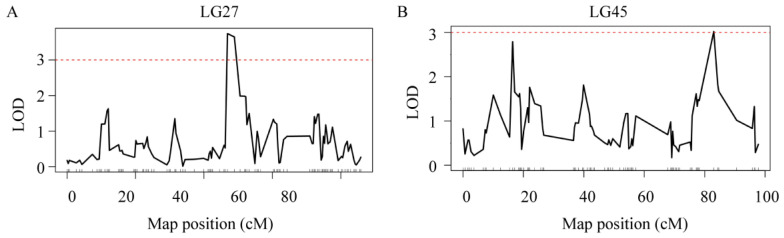
LOD curve of QTLs affecting the blood concentration of ammonia-N. The X-axis represents the marker distance, the Y-axis represents the LOD value, and the dashed line represents the delimited LOD threshold equal to 3. (**A**) Ammonia-N LOD curve on LG27. (**B**) Ammonia-N LOD curve on LG45.

The results of the QTL analysis and genetic effect estimation were shown that there were two ammonia-N tolerance sites on LG27 and LG45, and one urea-N tolerance site on LG27. We named the ammonia-N tolerance sites qAN1 and qAN2 and the urea-N tolerance sites qUN1. For ammonia-N, two QTLs were detected (Figure 2). qAN1 was located between 48.9 cM and 51.4 cM of LG27 (Figure 3A), with an LOD of 3.63. qAN2 was located between 83 cM and 83.4 cM of LG45 (Figure 3B), with an LOD of 3.02.

**Figure 4:**
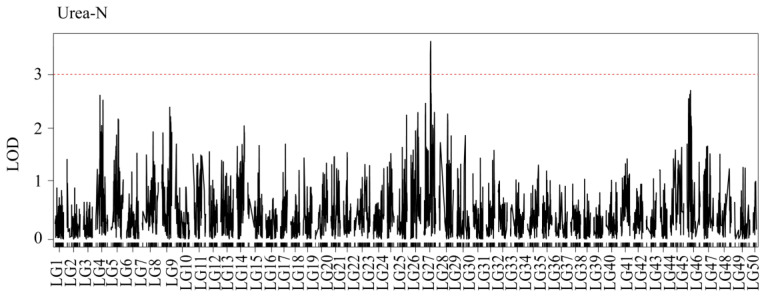
QTL mapping and significant regions were identified for the concentration of urea-N. The red dashed line represents the delimited LOD threshold equal to 3.

**Figure 5:**
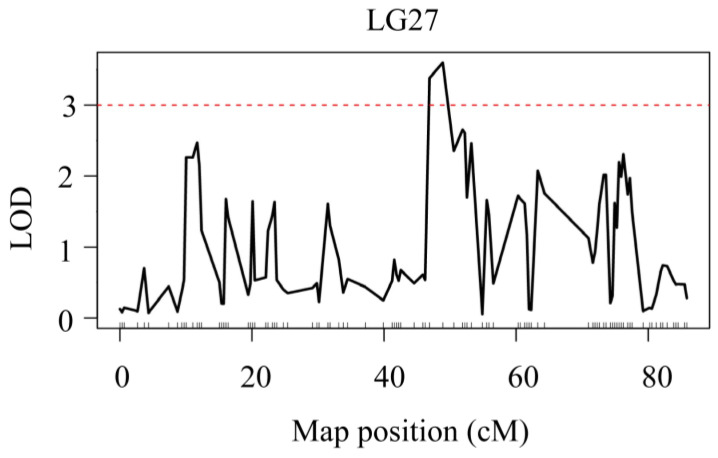
LOD curve of QTL affecting the blood concentration of urea-N on LG27. The X-axis represents the marker distance, the Y-axis represents the LOD value, and the red dashed line represents the delimited LOD threshold equal to 3.

For urea-N, one QTL, qUN1, was detected (Figure 4). Interestingly, qUN1 was also located between 48.9 and 51.4 cM of LG27 (Figure 5), and its LOD was 3.52. The levels of ammonia-N and urea-N were determined by different experimental methods, but the alkali tolerance traits were located on the same gene of the same chromosome, which indicated that the gene corresponding to the QTL might be strongly correlated with the high-alkali tolerance trait. Although qAN2 was also a QTL for the alkali-tolerant trait, its LOD value was very close to the significance threshold. Whether the gene corresponding to this QTL is related to the alkali-tolerant trait needs further verification. The traits of the QTL associated with alkali tolerance are presented in Table 2.

### 3.5. The Genotype Effect of QTL Peak

**Figure 6:**
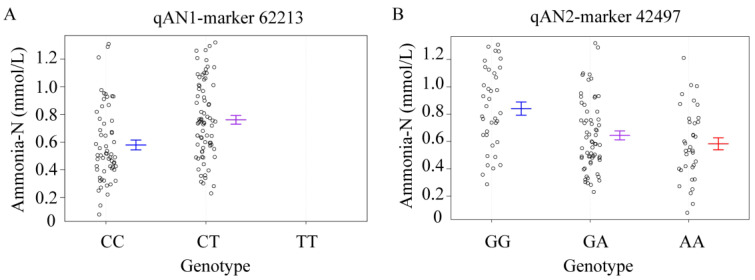
The QTL peak genotype effects of ammonia-N-related genes. The X-axis represents the marker genotype; the Y-axis represents the concentrations of ammonia-N. (**A**) qAN1 marker 62213. (**B**) qAN2 marker 42497. Blue, purple, and red bars represent means and error bars of different genotypes.

To investigate the genotype effect of QTL peak, we genotyped F1 hybrid progeny by SNP markers. For snp62213, CC was the alkali-tolerant homozygote, CT was the alkali-tolerant heterozygote, and TT was the alkali-non-tolerant homozygote. The numbers of the three genotypes were 72, 82, and 0, respectively. The blood ammonia-N contents of the CC and CT genotypes were 0.578 ± 0.271 mmol/L and 0.767 ± 0.273 mmol/L, respectively (Figure 6A). There were significant differences in blood ammonia content between CC and CT (*p* = 9.18 × 10^−5^). For snp42497, AA was the alkali-tolerant homozygote, GA was the alkali-tolerant heterozygote, and GG was the alkali-non-tolerant homozygote. The numbers of the three genotypes were 37, 69, and 48, respectively. The blood ammonia-N contents of AA, GA, and GG genotypes were 0.837 ± 0.300 mmol/L, 0.645 ± 0.262 mmol/L, and 0.607 ± 0.269 mmol/L, respectively (Figure 6B). There were significant differences in blood ammonia content between GG and GA (*p* = 6.00 × 10^−4^).

**Figure 7:**
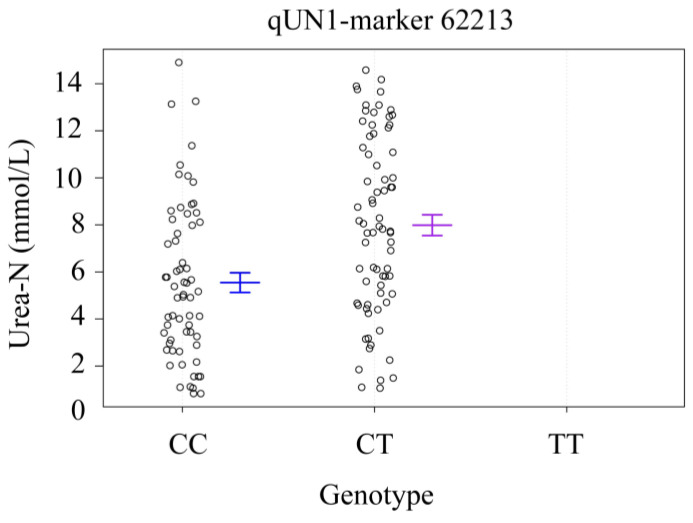
The QTL peak genotype effects of the urea-N-related gene. The X-axis represents the marker genotype; the Y-axis represents the concentrations of urea-N. Blue and purple bars represent means and error bars of CC and CT genotypes.

For qUN1, CC was the alkali-tolerant homozygote, CT was the alkali-tolerant heterozygote, and TT was the alkali-non-tolerant homozygote. The numbers of the three genotypes were 72, 82, and 0, respectively. The blood urea-N contents of CC and CT were 5.550 ± 3.320 mmol/L and 7.997 ± 3.770 mmol/L, respectively (Figure 7). There were significant differences in blood ammonia content between CC and CT (*p* = 1.15 × 10^−4^).

### 3.6. Candidate Genes Associated with Alkali-Tolerant Traits

**Figure 8:**
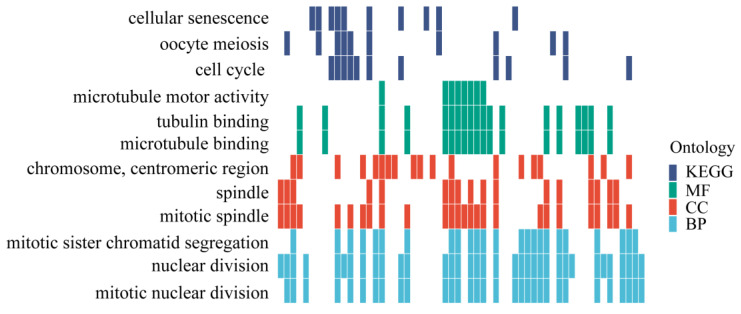
Go and KEGG pathway enrichment of potential alkali tolerance genes. Sky blue represents biological processes, red represents cellular components, green represents molecular functions, and dark blue represents the KEGG signaling pathway.

To further investigate the candidate genes associated with alkali-tolerant traits, we screened the genomic information and identified 586 potential alkali tolerance genes with SNP markers. These genes were found to be homologous to genes in humans; ggplot2 (version 3.5.0) was used for enrichment analysis. GO functional annotation revealed that these genes were involved in tubulin binding, mitotic spindle, nuclear division, and other processes. KEGG enrichment indicated that these genes may be involved in the cell cycle, cellular senescence, and oocyte meiosis signaling pathways (Figure 8).

**Figure 9:**
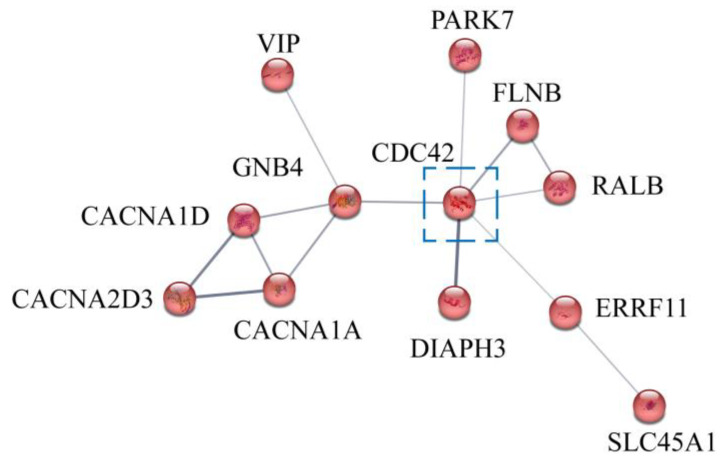
Interaction network of potential alkali tolerance genes on LG27.

To further identify potential alkali tolerance genes, we searched for candidate genes through genomic annotation and collected protein-coding genes at the nearest SNP site of each QTL. Two alkali-related genes were identified from the three QTLs. We identified one gene, cell division cycle 42 (CDC42), from qAN1 and qUN1 on LG27. The protein interaction network results showed that CDC42 was the key node of the interaction network, which suggested that CDC42 plays an important role in alkali-tolerant traits (Figure 9). Then, the signal pathway of CDC42 in the protein interaction network was analyzed, and the results showed that CDC42 participates in the G β: γ signaling through CDC42, the MAPK signal pathway, the EGF/EGFR signaling pathway, and the Ras signaling pathway (Table 3).

## 4. Discussion

The severity alkalization of environment caused by human activities and drought is increasing all over the world. Alkalization has occurred on 970 million hectares of land in at least 100 countries and is expected to continue at a rate of 1.5–3% per year [22,29]. In China, 99.13 million hectares of land have been alkalized, including 45.87 million hectares of alkaline lakes, which account for about 55% of the total lake area [30]. At the same time, problems such as environmental degradation, reduction of cultivated land area, and global warming have deeply affected the growth of food production [31,32]. Therefore, making full use of these alkaline lakes to develop aquaculture will greatly improve the food shortage problem. At present, only less than 2% of low-alkalinity water with alkalinity of 10 mmol/L has been developed and utilized by transplanting and domesticating conventional freshwater and seawater aquatic economic animals. However, the high pH (9–10) of these alkali lands will lead to metabolic alkalosis of aquatic organisms, which seriously affects the growth and reproduction of fish [33]. Lake Dali Nur crucian carp can naturally survive in such extreme environments and have become an important source of income for local natives. To understand the molecular mechanism of alkali adaptation of these natural alkali-tolerant fishes is the basis of the research of breeding species suitable for medium- and high-alkalinity saline-alkali waters.

The increase of exogenous ammonia-N concentration and the obstruction of endogenous urea-N of metabolic transformation will lead to the obstruction of ammonia excretion. The tolerance to alkali environment can be largely understood as the exclusion and tolerance of toxic substances such as ammonia-N and urea-N. The relationships between fish tolerance to high alkalinity, blood ammonia, and urea nitrogen were mainly reflected in the physiological adaptation mechanism to high-alkaline environments [34]. Under normal circumstances, the content of blood ammonia is extremely low and controlled within a normal range to maintain the health of fish [11]. However, under high-alkaline conditions, metabolic processes may change, affecting the production and clearance of blood ammonia, which in turn affects the ability to tolerate high alkalinity [35,36,37,38].

By studying the QTLs of Crucian carp, we can gain a deeper understanding of the genetic basis of alkali-tolerant traits [39,40,41,42,43]. The localization of QTLs not only helps to understand the genetic mechanisms of these traits but also provides important information for subsequent molecular-marker-assisted breeding [44,45,46,47,48,49]. Quantitative traits are caused by the combined effects of many minor genes. Usually, quantitative traits are the cumulative effects of multiple minor genes, which is a great challenge to finding trait loci. At the same time, tolerance genes tend to exhibit low heritability, and the genetic effects of these genes are not unstable in the offspring [43]. This adds to the uncertainty and difficulty of breeding. In this study, we used hybrid individuals for the study instead of comparing pure wild-type and domesticated types. Firstly, there are huge differences in living environment between wild-type crucian carp in Lake Dali Nur and domesticated bred red crucian carp, which may affect the performance in the alkali tolerance trait, making the comparison results less accurate. The hybrid individuals have heterosis, and we can obtain individuals with the alkali tolerance trait in the same environment. Secondly, the alkali tolerance trait is characterized by low heritability, and in order to enhance the likelihood of detecting QTLs associated with alkaline tolerance, the hybrid individuals were combined with different genetic backgrounds. The increase of genetic variation of the alkali tolerance trait in hybrid offsprings provide a possibility for QTL detection. Thirdly, by mating two different hybrid individuals, the interaction between loci and recessive genes can be studied, which is very important for the discovery of alkali tolerance genes. Finally, the hybrid individuals inherit genetic information from two different parents and obtain higher genetic diversity. The quantity and quality of alkali tolerance population can be effectively improved.

In this study, high-resolution genetic linkage maps enabled us to precise locate QTL for alkali-related traits. QTL analysis was performed for alkali-related traits based on the high-resolution linkage map. QTLs (qAN1 and qAN2) located between 48.9 cM and 51.4 cM on LG27 and between 83 cM and 83.4 cM on LG45 for the ammonia-N tolerance trait were detected, and qUN2 located between 48.9 cM and 51.4 cM for the urea-N tolerance trait was detected on LG27. This indicates that qAN1 and qUN1 correspond to the same gene, and this gene can tolerate both ammonia-N and urea-N. We successfully identified the locus of high-alkaline tolerance, which laid a solid foundation for the study of the molecular mechanism of high-alkaline tolerance. We genotyped grouped F1 hybrid progeny by SNP markers. It was found that under the condition of high alkali, the blood ammonia content of CC homozygous type was significantly lower than that of CT heterozygous type, and no TT genotype individuals were found. This indicates that the endogenous blood ammonia concentration of Lake Dali Nur crucian carp can be maintained at a relatively low level in a high-alkali environment to maintain the acid–base balance. The blood ammonia content of red crucian carp increased in the high-alkali environment, and the acid–base balance causes disorder, resulting in alkali poisoning. On the other hand, the urea-N content of CC homozygous type was significantly lower than that of CT heterozygous type, which indicated that the alkali tolerance of Lake Dali Nur crucian carp was mainly achieved by improving the alkali tolerance.

To further investigate the candidate genes, we identified 586 potential alkali tolerance genes using SNP markers. Of these genes, 148 are located on LG27, of which 61 are related to blood ammonia-N and 87 are related to urea-N. The protein interaction network analysis of these genes showed that CDC42 was the key node. At the same time, when we searched for candidate genes using genomic annotation and SNP markers, we found that qAN1 and qUN1 on LG27 are highly coincident, and they are both located in the intron of cell division cycle 42 (CDC42). This suggests that CDC42 was the most relevant gene to alkali tolerance among these genes. CDC42 is a guanine nucleotide exchange factor (GEF) that can switch between active and inactive forms, serving as a molecular switch to perform various important regulatory functions. In this study, KEGG signal pathway analysis results showed that CDC42 participated in the G β: γ signaling through CDC42, the MAPK signal pathway, the EGF/EGFR signaling pathway, and the Ras signaling pathway. Among the four signaling pathways, Gbeta detects extracellular signals and activates downstream signaling pathways. Similarly, the Ras signaling pathway is involved in the transmission of intracellular signals to control downstream cell proliferation, survival, growth, migration, and differentiation. The MAPK signaling pathway and EGF/EGFR signaling pathway are both downstream effect pathways. The MAPK signaling pathway is involved in cell proliferation, differentiation, and migration, playing an important role in response to pro-inflammatory stimuli. The EGF/EGFR signaling pathway can induce cell growth, differentiation, migration adhesion, and cell survival by activating extracellular signaling regulating kinases and activating many transcriptional regulatory factors [50,51,52]. This suggests that CDC42 may play an important role in alkali tolerance.

## 5. Conclusions

In conclusion, we conducted a comprehensive mapping of QTLs for alkaline tolerance in crucian carp using genome-wide SNP markers. The genotype effect of the QTL peak led to the identification of several specific characteristic genes related to blood ammonia-N and urea-N. Genomic annotation revealed CDC42 as the candidate functional gene, functionally associated with G β: γ signaling through CDC42, the MAPK signal pathway, the EGF/EGFR signaling pathway, and the Ras signaling pathway. This study has enhanced our understanding of the alkali tolerance of crucian carp in extremely alkaline environments and has laid a foundation for future MAS breeding in alkaline and saline lake aquaculture.

## Figures and Tables

**Table 1:** Summary of phenotypic data of alkali-related traits in the full-sib family.

Trait	Minimum	Maximum	Mean ± SD	Coefficient of Variation
Ammonia-N	0.827	14.903	6.856 ± 0.093	0.418
Urea-N	0.143	1.308	0.681 ± 0.166	0.547

**Table 2:** Analysis of three QTLS and evaluation of genetic effects of the two alkali-related traits.

Trait	Linkage Group	QTL Name	Linkage Group Size (cM)	LOD	Var	Nearest Marker
Ammonia-N	LG27	qAN1	48.9	3.63	1.14	snp62213
Ammonia-N	LG45	qAN2	83	3.02	6.75	snp42497
Urea-N	LG27	qUN1	48.9	3.52	1.88	snp62213

**Table 3:** Enriched gene pathways in the QTL regions.

Term ID	Term Name	Genes in Term
HAS-896461	G β γ signaling through CDC42	CDC42 GNB4
WP382	MAPK signaling pathway	CDC42 FLNB
WP437	EGF/EGF2R signaling pathway	CDC42 RALB ERRF11
WP4223	Ras signaling pathway	CDC42 RALB GNB4

## Data Availability

The datasets presented in this study can be found in online repositories. The names of the repository/repositories and accession number(s) can be found below: https://ngdc.cncb.ac.cn/gsa/s/EV0l2Aaq, accessed on 23 April 2024.

## Supplementary Materials

The following supporting information can be downloaded at https://www.mdpi.com/article/10.3390/genes15060751/s1. Table S1: Summary of the linkage map.
